# The Impact of Leadership in Gender Diversity of Annual Meeting Speakers in the American Society for Surgery of the Hand

**DOI:** 10.1016/j.jhsg.2025.100823

**Published:** 2025-09-13

**Authors:** Mehrdad Farrokhi, Mohammad Amin Nochian

**Affiliations:** ∗ Eris Research Institute, Tehran, Iran; † Student Research Committee, Faculty of Nursing and Midwifery, Semnan University of Medical Sciences, Semnan, Iran

To the Editor:

We read with great interest the recently published article by Kim et al^1^ in the *Journal of Hand Surgery Global Online*. The main aim of this study was to compare the gender and racial diversity of speakers and moderators at the American Society for Surgery of the Hand annual meetings in 2022 and 2023, coinciding with a leadership transition from men to women. The authors collected data on the number of speaking opportunities and assessed the gender and race of each speaker based on perceived visual perceptions from photographs.

Unexpectedly, χ^2^ tests were employed to compare the diversity metrics between the 2 years. It is important to note that the χ^2^ test is generally used to compare categorical variables between independent groups.^2^^,^^3^ Given that this analysis involves comparisons of the same cohort over two time points, we suggest that the authors consider using the McNemar test for a more accurate analysis of the paired nominal data.^4^

## Conflicts of Interest

No benefits in any form have been received or will be received related directly to this article.

**Using artificial intelligence chatbots**

The authors used AI-powered tools to check grammar and enhance the academic quality of the text, which was primarily written by the authors.
